# Plant biology research at BGRS-2018

**DOI:** 10.1186/s12870-019-1634-0

**Published:** 2019-02-15

**Authors:** Yuriy L. Orlov, Elena A. Salina, Gilda Eslami, Alex V. Kochetov

**Affiliations:** 1 Institute of Cytology and Genetics SB RAS, 630090 Novosibirsk, Russia; 2 Novosibirsk State University, 630090 Novosibirsk, Russia; 3 Research Center for Food Hygiene and Safety, Department of Parasitology and Mycology, Shahid Sadoughi University of Medical Sciences, Yazd, Iran

This thematic issue of BMC Plant Biology contains materials from the bioinformatics and systems biology multi-conference BGRS\SB-2018 (Bioinformatics of Genome Regulation and Structure\Systems Biology - 2018), traditional biannual event in Novosibirsk, Russia (http://conf.bionet.nsc.ru/bgrssb2018/en/). This Special Issue is accompanied by other BioMed Central Special Issues collecting papers in the fields of bioinformatics, systems biology genomics, evolutionary biology and genetics, presented at BGRS event. BMC Genomics, BMC Evolutionary Biology, BMC Systems Biology and BMC Plant Biology Supplements had covered previous BGRS-2016 materials [1–4], as well as works discussed at Belyaev Conference-2017 (http://conf.bionet.nsc.ru/belyaev100/en) and published at BioMed Central special journal issues [5–7]. BGRS\SB-2018 included several symposia and sessions on computational plant biology.

Current post-conference journal issue shows bioinformatics and genomics approaches in crop plant biology. Here is a brief summary of the papers in this issue.

The paper by Alina E. Dresvyannikova et al. [8] (this issue) opens the journal issue by presenting research on one of the most important agricultural species - bread wheat. Leaves of related species have a unique morphological feature: they consist of a proximal sheath and a distal blade separated by a ligular region. The authors characterized an induced liguleless mutant (LM) of *Aegilopstauschii*Coss., a donor of genome D of bread wheat *Triticumaestivum* L. Liguleless variants have an upright leaf blade that wraps around the culm. Research on liguleless mutants of maize and other cereals has led to identification of genes that are involved in leaf patterning and differentiation. Dresvyannikova and co-authors report characterization of a liguleless*Ae. tauschii* mutant, whose phenotype is under control of a dominant mutation of Lgt. The dominant mode of inheritance of the liguleless trait in a Triticeae species is reported for the first time. The characterized Lgt mutant represents a new model for further investigation of plant leaf patterning and differentiation.

Note, this work continues series of publications in previous BMC Plant Biol special issues on search of differentiation of floral meristems in wheat [9]. Earlier FRIZZY PANICLE gene was shown as driver gene for supernumerary spikelets in bread wheat [10].

Ksenia V. Strygina and Elena K. Khlestkina [11] continue topic of gene regulation in wheat discussing structural and functional organization of Myc-like transcriptional factors. Myc-like regulatory factors carrying the basic helix–loop–helix (bHLH) domain belong to a large superfamily of transcriptional factors (TFs) present in all eukaryotic kingdoms. In plants, the representatives of this superfamily regulate diverse biological processes including growth and development as well as response to various stresses. As members of the regulatory MBW complexes, they participate in biosynthesis of flavonoids. In wheat, only one member (*TaMyc1*) of the Myc-like TFs family has been studied, while structural and functional organization of further members remained uncharacterized. Myc-like co-regulator (*TaMyc-B1*) of anthocyanin synthesis in wheat coleoptile was described for the first time.

Elena I. Gordeeva and co-authors [12] (this issue) present analysis of the anthocyanin biosynthesis regulatory network in barley, another important crop plant. Anthocyanins are flavonoid pigments that accumulate in vacuoles of a wide range of plant cells and tissues. They are responsible for orange, brown, red, blue and purple colors in vegetative and reproductive parts of plants. The scientific interest to the pigments and their uncolored precursors has been attracting by their important functions in plant physiology and human health promoting effects. In plants overall, bHLH regulates anthocyanin biosynthesis together with TF harboring R2R3-MYB domain. In wheat, the R2R3-MYBs responsible for purple color of grain pericarp are encoded by the homoallelic series of the Pp-1 genes that were mapped on the short arms of chromosomes 7. In barley, in orthologous positions to wheat’s Pp-1, the Ant1 gene determining red color of leaf sheath has been mapped. In the current study, the authors tested whether Ant1 has pleiotropic effect not only on leaf sheath color but also on pericarp pigmentation. The R2R3-MYB-encoding counterpart (Ant1) of the regulatory Ant2 gene was determined for the first time. The dominant alleles of the both of them are required for activation of anthocyanin synthesis in barley lemma and pericarp. Earlier the interaction between the anthocyanin biosynthesis regulatory genes has been revealed in dicot plant species only. Gordeeva et al. demonstrated that the regulatory mechanism is considered to be more common for plant kingdom.

Maksim S. Makarenko and colleagues [13] (this issue) analyzed genomics data for another important plant species - sunflower - characterizing the mitochondrial genome of the MAX1 type. More than 70 cytoplasmic male sterility types (CMS) have been identified in Helianthus, but only for less than half of them, research of mitochondrial organization has been conducted. In the present study, the authors have investigated structural changes in the mitochondrial genome of HA89 (MAX1) CMS sunflower line in comparison to the fertile mitochondrial genome.

Alexey A. Dmitriev [14](this issue) studied flax (*Linumusitatissimum* L.) stress-response genes. Flax (*Linumusitatissimum* L.) is grown for fiber and seed production. Understanding the mechanisms of flax response to the stresses and identification of resistance gene candidates will help in breeding of improved cultivars. The response of flax plants to increased pH level and zinc (Zn) deficiency was studied. We identified genes with expression alterations in flax under non-optimal soil acidity and Zn deficiency based on high-throughput sequencing data. These genes are involved in diverse processes, including ion transport, cell wall biogenesis, and photosynthesis, and could play an important role in flax response to the studied stresses. Moreover, genes with distinct expression changes between examined tolerant and sensitive genotypes could determine the mechanisms of flax tolerance to non-optimal acidity and Zn deficiency.

Anna Klepikova et al. [15] (this issue) present an update to the database TraVA on transcriptome maps in plants. Transcriptome maps that include different organs, tissues, cells and stages of development are currently available for at least 30 plants. However, most studies explore only limited set of organs and developmental stages (leaves or seedlings). In order to provide broader view of organ-specific strategies of cold stress response Klepikova and colleagues studied expression changes that follow exposure to cold (+ 4 °C) in different aerial parts of plant: cotyledons, hypocotyl, leaves, young flowers, mature flowers and seeds using RNA-seq. The results were integrated with previously published transcriptome map of *Arabidopsis thaliana* and used for an update of a public database TraVa: http://travadb.org/.

Alexey V. Doroshkov [16] discussed evolution of gene regulatory networks controlling trichome development in plants. Natural variety of trichomes and their accessibility makes them a fruitful model for studying the molecular processes of cell fate determination, cell cycle control and cellular morphogenesis. Nowadays, a large number of genes regulating the morphogenesis of trichomes for *A. thaliana* are described. The study was aimed on the evolution of the trichomes formation gene regulatory networks that is also used in other developmental processes. The results allowed hypothesizing that divergence and/or specialization of the trichomes formation gene regulatory network components associated with origination of plant taxa. In addition, a number of candidate genes responsible for the development of trichomes in a wide range of species were predicted.

Therefore, this issue includes reports of recent bioinformatics application in computational plant biology.

BGRS\SB-2018 multi-conference had several parallel symposia, sessions and workshops, including First Sino-Russian Workshop on Integrative Bioinformatics and Systems Biology (http://conf.bionet.nsc.ru/srw2018/en/) and international Round table on education in bioinformatics. Other related computational biology works are presented in parallel BioMed Central issues by 2018. We invite our readers worldwide to attend our next events - Systems Biology and Bioinformatics Young Scientists School in summer 2019 and PlantGen-2019 conference (http://conf.bionet.nsc.ru/plantgen2019/en/) in Novosibirsk, Russia.
